# Inactivated COVID-19 Vaccine Induces a Low Humoral Immune Response in a Subset of Dermatological Patients Receiving Immunosuppressants

**DOI:** 10.3389/fmed.2021.769845

**Published:** 2021-12-08

**Authors:** Chutima Seree-aphinan, Kumutnart Chanprapaph, Ploysyne Rattanakaemakorn, Chavachol Setthaudom, Thanitta Suangtamai, Cherrin Pomsoong, Yanisa Ratanapokasatit, Poonkiat Suchonwanit

**Affiliations:** ^1^Division of Dermatology, Department of Medicine, Faculty of Medicine, Ramathibodi Hospital, Mahidol University, Bangkok, Thailand; ^2^Immunology Laboratory, Department of Pathology, Faculty of Medicine, Ramathibodi Hospital, Mahidol University, Bangkok, Thailand; ^3^Division of Allergy, Immunology, and Rheumatology, Department of Medicine, Faculty of Medicine, Ramathibodi Hospital, Mahidol University, Bangkok, Thailand

**Keywords:** autoimmune skin diseases, Sinovac, CoronaVac, inactivated COVID-19 vaccine, immunosuppression, immunogenicity

## Abstract

Inactivated Sinovac-CoronaVac vaccine (Sinovac Life Sciences, Beijing) for coronavirus disease 2019 (COVID-19) has been used in many countries. However, its immunogenicity profile in immunosuppressed dermatological patients is lacking. This prospective observational case-control study compared the humoral immune response between adult dermatological patients receiving systemic immunosuppressive therapies (*n* = 14) and those who did not (*n* = 18); excluding patients with HIV infection, cancer, non-dermatological autoimmune conditions, previous COVID-19 infection, and positive anti-severe acute respiratory syndrome coronavirus 2 (SARS-CoV-2) IgG prior to vaccination. The subjects were advised to withhold methotrexate for 1 week after each vaccine dose while continuing other therapies unadjusted. Anti-SARS-CoV-2 IgG antibody, surrogate neutralizing antibody (sNAb), and seroconversion rates (calculated from the percentages of participants in the group with positive sNAb) were used to assess immunogenicity. We found that participants using azathioprine, cyclosporin, mycophenolate mofetil, or prednisolone ≥ 10 mg/day had a lower level of serum anti-SARS-CoV-2 IgG antibody and sNAb than those received methotrexate ≤ 10 mg/week, prednisolone < 10 mg/day, or biologics (i.e., secukinumab, ixekizumab, omalizumab). Patients who received methotrexate ≤ 10 mg/week, prednisolone < 10 mg/day or the biologics had a similar immunogenicity profile to those without immunosuppressive therapies. Despite the lack of statistical significance, a reduction of humoral immune response was observed among the study participants who used ≥2 immunosuppressants or pemphigus patients. Our findings suggest that a subset of patients with immune-mediated skin conditions respond poorly to the vaccine despite having low-level immunosuppression. These patients could benefit from vaccines that trigger a greater level of immunogenicity or booster doses.

## Introduction

Coronavirus Disease 2019 (COVID-19) is a global health emergency; the disease has cost millions of lives and greatly disrupt the world economy (1). Vaccination is the key to ameliorating the situation and potentially stopping this ongoing pandemic, as evidence has shown a decline of new and hospitalized COVID-19 cases in countries with high vaccination coverage (2). Inactivated Sinovac-CoronaVac vaccine (Sinovac Life Sciences, Beijing) has been used for mass vaccination in many countries as it is proven to give an acceptable level of protection against symptomatic and severe COVID-19 disease in volunteers (3). However, data regarding the vaccine's immunogenicity in autoimmune dermatological patients is not available, making it challenging to recommend whether the vaccine is suitable for these patients.

Currently, the available evidence of inactivated COVID-19 vaccine is limited to the groups of autoimmune rheumatic disease (ARD) patients, which suggests a reduced but acceptable level of the vaccine's immunogenicity (4, 5). Nevertheless, it is not possible to assume that immunosuppressed dermatological patients will have the same immunogenicity profile, as there is only a partial overlap within the disease spectrum of autoimmune rheumatologic and dermatologic conditions. Moreover, different pathogenesis leads to a distinct treatment approach and immunosuppressive agents used. To fill in this gap of knowledge, this study aims to compare humoral immune responses after a complete course of inactivated Sinovac-CoronaVac COVID-19 vaccine (referred to as CoronaVac) between adult dermatological patients receiving systemic immunosuppressive therapies and those who did not.

## Methods

### Study Design and Participants

This is a prospective observational case-control study conducted in a university hospital's dermatology outpatient clinic. The study was approved by the Human Research Ethics Committee, Faculty of Medicine Ramathibodi Hospital, Mahidol University (MURA 2021/446). Patients scheduled to receive the CoronaVac vaccine distributed *via* Thailand's national vaccination scheme were screened for eligibility. Under this scheme, the vaccine was administered in two 3-μg doses, 3–4 weeks apart.

Patients with immune-mediated dermatological conditions who had been treated with systemic immunosuppressive agents from 1 month before to 1 month after vaccination were recruited as cases. Individuals who did not receive systemic immunosuppressive agents were recruited as controls. In patients who had been treated with rituximab, they were categorized as cases or controls based on their post-rituximab B cell status. Those whose B cells were depleted (CD19^+^ lymphocyte < 5%) were categorized as cases. Those whose B cells were incompletely depleted or repopulated after rituximab therapy (CD19^+^ lymphocyte ≥ 5%) were also defined as cases when an additional immunosuppressant is needed for disease control. These cases were classified according to their current medication. However, those who achieved complete B cell repopulation, but remained in complete remission without treatment, were classified as controls. The patients with HIV infection, cancer, non-dermatological autoimmune conditions, history of previous COVID-19 infection, positive for anti-severe acute respiratory syndrome coronavirus 2 (SARS-CoV-2) IgG prior to vaccination were excluded. According to the current recommendation (6), the subjects were advised to temporarily withhold methotrexate for 1 week after each vaccine dose while continuing other therapies unadjusted.

### Immunogenicity Assessment

The magnitude of humoral immune responses was assessed using the serum levels of anti-SARS-CoV-2 IgG antibody and SARS-CoV-2 surrogate neutralizing antibody (sNAb). Three milliliters of whole blood were collected from each participant before vaccination and 4 weeks after receiving the second dose of the vaccine. The samples were stored in clot activator tubes (VACUETTE^®^, Greiner Bio-One, Austria) and allowed to clot at room temperature for at least 10–15 min before centrifuging at 3,500 g for 10 min. The serums retrieved from the supernatant were transferred to 1.5-mL sterile polypropylene tubes using Pasteur pipettes and stored at −20°C until analysis. None of the serum samples was hemolyzed, icteric, or lipemic. All serum samples were thawed once with the storage time before an analysis between 2 and 4 weeks.

Serum anti-SARS-CoV-2 IgG antibody level was quantified, before and after vaccination, using automated chemiluminescent microparticle immunoassay (Abbott Laboratories, United States), which reports the concentration of serum anti-SARS-CoV-2 IgG antibody level in an arbitrary unit (AU) with a positive cut-off threshold recommended by the manufacturer at 50 AU/milliliters. Surrogate virus neutralization assays (SARS-CoV-2-NeutraLISA, Euroimmun, Germany) were performed on post-vaccination serum samples to measure the amount of sNAb in the form of the neutralizing activity. The tests were executed per the manufacturer's instructions by trained laboratory personnel; the system reports neutralizing activity of sNAb as the percentage of inhibition. The positive cut-off threshold recommended by the manufacturer is 35%. We did not perform the surrogate virus neutralization assay on pre-vaccinated serum samples as they were negative for anti-SARS-CoV-2 IgG antibody.

### Data Collection and Statistical Analysis

#### Baseline Characteristics

For all study participants, age, sex, and the diagnosis of skin diseases were collected. In patients who received immunosuppressive agents, the number, name, and dosage were documented. Baseline serum IgA, IgM, IgG levels, as well as the percentages of circulating CD19^+^ B lymphocytes, CD4^+^ T lymphocytes, and CD8^+^ T lymphocytes (quantified by routine flow cytometry analysis), were measured. Baseline characteristics, serum anti-SARS-CoV-2 IgG antibody level, and neutralizing activity of sNAb were compared between cases and controls. In addition, subgroup analyses were explored in patients with different skin conditions and different types of immunosuppressants used. Fisher's exact tests were employed to compare categorical variables. The between-group comparisons of normally distributed and non-normally distributed continuous variables were performed with *t*-tests and Wilcoxon rank-sum tests, respectively.

#### Study Outcomes

The outcome measures were serum anti-SARS-CoV-2 IgG levels, neutralizing activity of sNAb, and seroconversion rates. Seroconversion rates were calculated from the percentages of participants in the group who tested positive for sNAb post-vaccination. We did not use anti-SARS-CoV-2 IgG levels to compute seroconversion rates because its level may partly represent a cross-reactivity of anti-SARS-CoV-2 IgG to endogenous antibodies and cross-antigens (7). The measured values were compared between cases and controls using *t*-tests (for data with normal distribution) and Wilcoxon rank-sum tests (for data with non-normal distribution). Comparisons of serum anti-SARS-CoV-2 IgG antibody level and neutralizing activity of sNAb among subgroups were made using one-way analysis of variance with *post-hoc* Bonferroni tests (for data with normal distribution) and Kruskal-Wallis tests with *post-hoc* Dunn's tests (for data with non-normal distribution). Seroconversions rates were compared between subgroups with Fisher's exact tests. Statistical analysis was performed with STATA 17.0 (StataCorp LLC, TX, US). The graphical illustrations were created with the R software version 4.1.0 (R Foundation for Statistical Computing, Vienna, Austria) using ggplot2 data visualization package. *P*-value < 0.05 was considered statistically significant.

## Results

Thirty-two patients who received a complete course of the CoronaVac vaccine were enrolled in the study. Baseline characteristics of the study participants are shown in Table 1. Fourteen patients with pemphigus, psoriasis and chronic spontaneous urticaria were recruited as cases. The participants who served as controls (mostly patients with acne, melasma, androgenetic alopecia, seborrheic keratosis) did not use topical or systemic immunosuppressive therapies. The cases and controls were age- and sex-matched. The medications used among the cases included azathioprine (25–150 mg/day), mycophenolate mofetil (1,000 mg/day), cyclosporin (50 mg/day), methotrexate (7.5–10 mg/week), prednisolone (5–20 mg/day), biologics (i.e., secukinumab, ixekizumab, and omalizumab at standard doses for their respective disorders); 42.8% of the patients received ≥ 2 of these medications. Three pemphigus cases had a history of rituximab use 2 years before recruitment; all had CD19^+^ B cells ≥ 5%. Two patients who were in complete remission under minimal adjuvant therapy were classified as cases, while one patient, who was in complete remission off therapy, was assigned to a control group. At baseline, flow cytometry studies for the number of circulating total lymphocytes, CD19^+^ B lymphocytes, CD4^+^ T lymphocytes, and CD8^+^ T lymphocytes and serum concentration of total immunoglobulins demonstrated comparable results between groups. Serum SARS-CoV-2 IgG antibody level was undetectable in the pre-vaccinated serum samples of both cases and controls. The most common side effects experienced among study participants were low-grade fever, myalgia, mild tenderness at the injection site, and somnolence. One psoriasis patient had a flare-up after vaccination, while the others remained under control.

**Table 1:** Baseline characteristics of the study participants.

**Characteristics**	**Case (*N* = 14) *n* (%)**	**Control (*N* = 18) *n* (%)**	***p*** **-value**
Age (mean, 95% CI)	43.9 (36.6–51.2)	44.6 (37.1–52.0)	0.890^a^
Sex, male (%)	4 (28.6)	7 (38.9)	0.712^b^
Autoimmune skin diseases (%)			<0.001^*^^b^
- Pemphigus	7 (50.0)	1 (5.6)	
- Psoriasis	6 (42.9)	1 (5.6)	
- Chronic spontaneous urticaria	1 (7.1)	1 (5.6)	
- No autoimmune skin diseases	0 (0)	15 (83.2)	
**Baseline peripheral blood flow cytometry** **(percentages among total lymphocytes, mean, 95%CI)**			
- %CD4^+^ T-lymphocytes	61.3 (56.6–66.0)	59.6 (55.1–64.0)	0.585^a^
- %CD8^+^ T-lymphocytes	31.1 (27.1–35.1)	30.8 (26.5–35.0)	0.902^a^
- %CD19^+^ B-lymphocytes	14.9 (11.6–18.1)	14.2 (11.7–16.7)	0.742^a^
**Baseline immunoglobulin level (g/L)**			
- IgM (median, IQR)	1.0 (0.6–1.8)	1.0 (0.7–1.5)	0.718^c^
- IgG (median, IQR)	12.5 (11.4–14.4)	12.8 (10.4–14.7)	0.909^c^
- IgA (mean, 95%CI)	2.9 (2.4–3.3)	2.4 (2.1–2.8)	0.082^a^
**Systemic immunosuppressive medications** **(% of participants who used the medications)**			
- Azathioprine	5 (35.7)	0	
- Cyclosporin	1 (7.1)	0	
- Mycophenolate mofetil	1 (7.1)	0	
- Moderate-to-high dose prednisolone (≥10 mg/day)	2 (14.3)	0	
- Low-dose prednisolone (<10 mg/day)	3 (21.4)	0	
- High-dose methotrexate (>10 mg/week)	0 (0)	0	
- Low-dose methotrexate (≤ 10 mg/week)	3 (21.4)	0	
- Biologics^‡^	6 (42.9)	0	
**Number of immunosuppressants used (%)**			
- 0	0 (0)	18 (100)	
- 1	8 (57.2)	0	
- 2	5 (35.7)	0	
- 3	1 (7.1)	0	
**Post-vaccination immunogenicity**			
- Anti-SARS-CoV-2 IgG (AU/mL, median, IQR)	666.2 (312.2–987.3)	1,208.0 (774.1–1,910.0)	0.028^*^^c^
- Neutralizing activity of sNAb (%inhibition, mean, 95%CI)	43.1 (29.2–57.0)	52.9 (41.3–64.6)	0.252^a^
- Post-vaccination seroconversion rate^†^ (%)	56.3	77.8	0.180^d^

† *Seroconversion rates were calculated from the percentages of study participants who tested positive for sNAb in the group*.

‡ *Biologics include secukinumab, ixekizumab, and omalizumab at standard doses for their respective disorders*.

a *p-value from t-tests*.

b *p-value from Fisher's exact tests*.

c *p-value from Wilcoxon rank-sum tests*.

d *p-value from Chi-squared tests*.

* *p < 0.05*.

*AU/mL, arbitrary unit per milliliter; CD, cluster of differentiation; CI, confidence interval; IgA, immunoglobulin A; IgG, immunoglobulin G; IgM, immunoglobulin M; IQR, interquartile range; sNAb, surrogate neutralizing antibody; SARS-CoV-2, severe acute respiratory syndrome coronavirus 2*.

**Figure 1:**
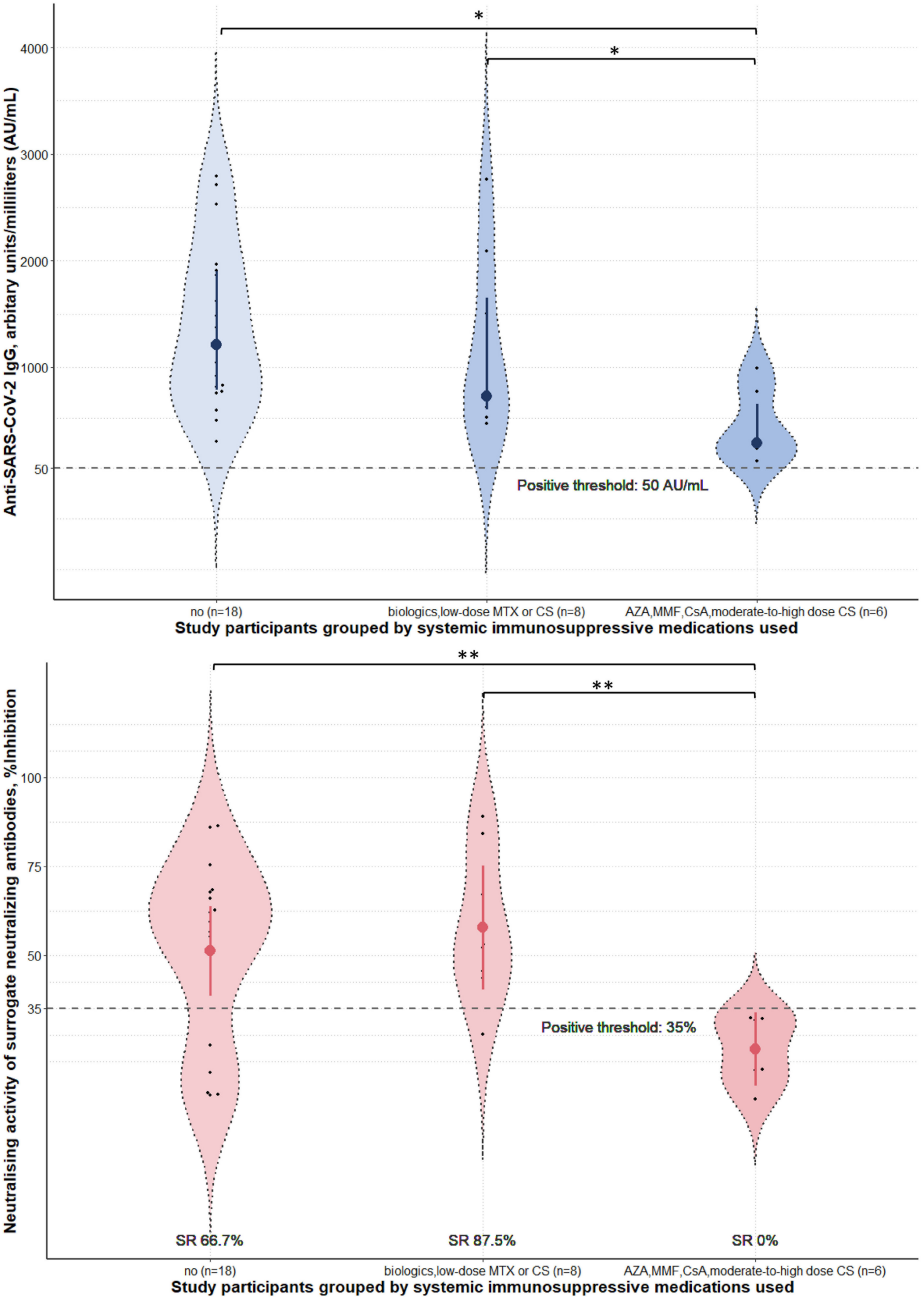
Immunogenicity of the inactivated Sinovac-CoronaVac COVID-19 vaccine in study participants stratified by the immunosuppressive medications used. Serum anti-SARS-CoV-2 IgG antibody level and neutralizing activity of surrogate neutralizing antibody were measured 4 weeks post-second dose of the vaccine. Data were presented with violin plots containing medians and interquartile range for anti-SARS-CoV-2 IgG antibody level and means and 95% confidence interval for neutralizing activity. Seroconversion rates (SR) for each subgroup were calculated from the percentages of study participants who tested positive for sNAb in the group. Prednisolone <10 mg and ≥10 mg were considered low-dose and moderate-to-high dose. Methotrexate ≤ 10 mg/week was defined as low-dose. AZA, azathioprine; CS, corticosteroids; CsA, cyclosporin; MTX, methotrexate; MMF, mycophenolate mofetil; SARS-CoV-2, severe acute respiratory syndrome coronavirus 2; SR, seroconversion rates. *p-value from Kruskal-Wallis tests *post-hoc* Dunn's tests < 0.05. **p-value from one-way analysis of variance with *post-hoc* Bonferroni tests < 0.05.

**Figure 2:**
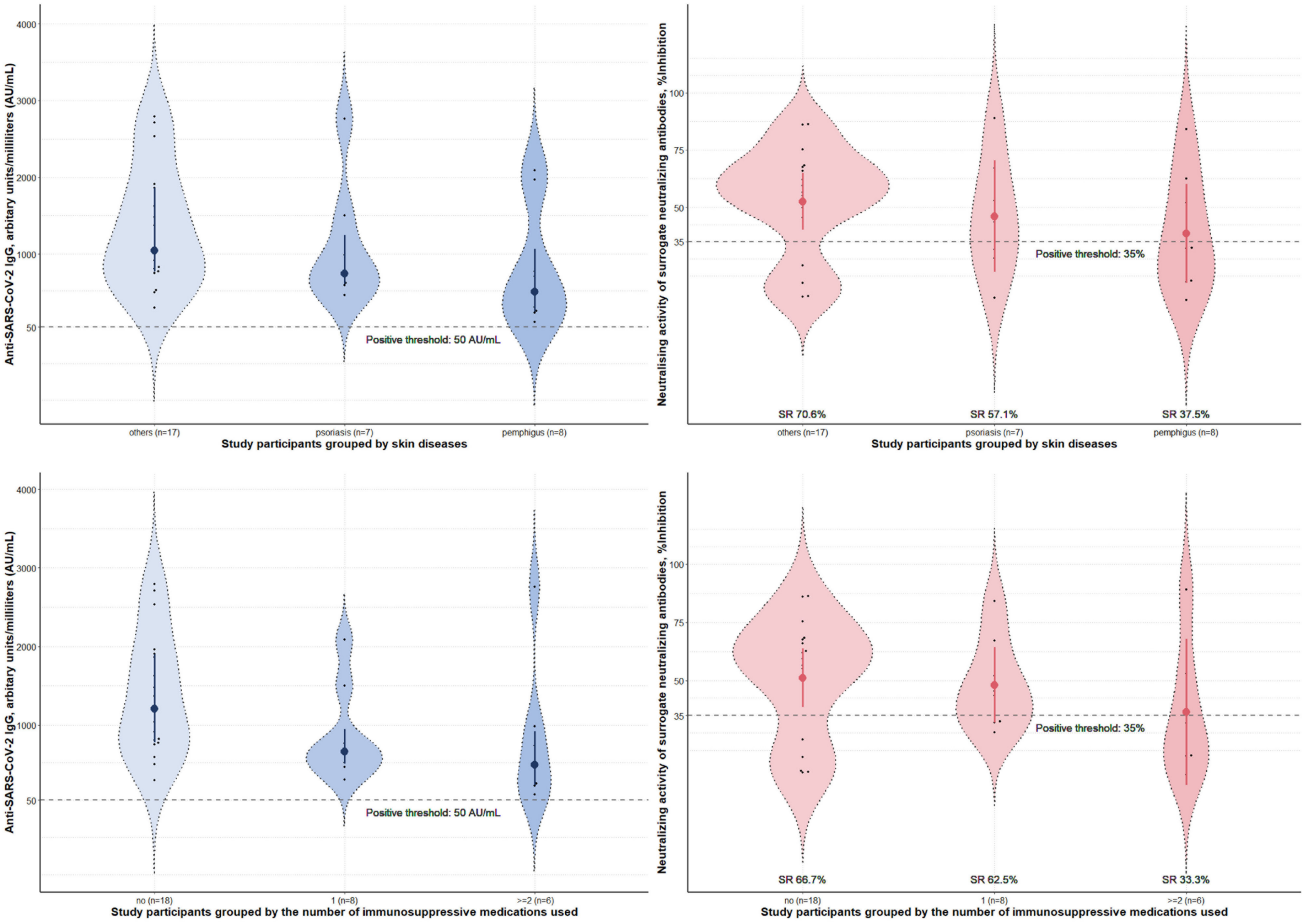
Immunogenicity of the inactivated Sinovac-CoronaVac COVID-19 vaccine in study participants stratified by skin diseases and the number of immunosuppressants used. Serum anti-SARS-CoV-2 IgG antibody level and neutralizing activity of surrogate neutralizing antibody measured 4 weeks post-second dose of the vaccine. Data were presented with violin plots containing medians and interquartile range for anti-SARS-CoV-2 IgG antibody level and means and 95% confidence interval for neutralizing activity. Seroconversion rates (SR) for each subgroup were calculated from the percentages of study participants who tested positive for sNAb in the group. No statistically significant difference was found between groups. SARS-CoV-2, severe acute respiratory syndrome coronavirus 2; SR, seroconversion rates.

Four weeks after the second vaccine dose, all participants had positive results of serum anti-SARS-CoV-2 IgG antibody. Albeit statistical insignificance, there was a trend toward lower sNAb levels and seroconversion rates in cases than controls (Table 1). Subgroup analyses showed that participants using azathioprine, cyclosporin, mycophenolate mofetil, or moderate-to-high-dose corticosteroids (prednisolone ≥ 10 mg/day) had a substantially lower neutralizing activity of sNAb than those who received low-dose methotrexate (≤10 mg/week), low-dose systemic corticosteroids (prednisolone < 10 mg/day), or the biologics (Figure 1). Moreover, none of the study participants within the former group developed seroconversion after vaccination (Table 2). In contrast, patients who received low-dose methotrexate, low-dose systemic corticosteroids, or the biologics had a similar immunogenicity profile to the study participants without immunosuppressive therapies. Despite the lack of statistical significance, a reduction of humoral immune responses was observed among participants who used ≥2 immunosuppressants. Likewise, pemphigus patients had lower humoral immune responses than other conditions, although this analysis did not reach a statistical significance level (Figure 2). None of the participants developed symptomatic COVID-19 infection during a minimum of 3-month follow-up period after vaccination.

**Table 2:** Subgroup analyses of seroconversion rates in patients stratified by skin diseases and the number of immunosuppressants used.

**Subgrouping by**	**Seroconversion**^†^ **(%row)**		***p*** **-value**
	**Yes *n* (%)**	**No *n* (%)**	
**Types of immunosuppressants used**			0.003^*^^a^
◾ Azathioprine, Cyclosporin, Mycophenolate mofetil, Prednisolone ≥ 10 mg/day.	0 (0)	6 (100.0)	
◾ Methotrexate ≤ 10 mg/week, Prednisolone <10 mg/day, Biologics^‡^	7 (87.5)	1 (12.5)	
◾ No immunosuppressants used	12 (66.7)	6 (33.3)	
**Skin diseases**			0.288^a^
◾ Pemphigus	3 (37.5)	5 (62.5)	
◾ Psoriasis	4 (57.1)	3 (42.9)	
◾ Others^#^	12 (70.6)	5 (29.4)	
**The number of immunosuppressants used**			0.347^a^
◾ 0	12 (66.7)	6 (33.3)	
◾ 1	5 (62.5)	3 (37.5)	
◾ ≥2	2 (33.3)	4 (66.7)	

† *Seroconversion rates were calculated from the percentages of study participants who tested positive for sNAb in the group*.

‡ *Biologics include secukinumab, ixekizumab, and omalizumab at standard doses for their respective disorders*.

# *Other diseases include chronic spontaneous urticaria, acne, melasma, androgenetic alopecia, and seborrheic keratosis*.

a *p-value from Fisher's exact tests*.

* *p < 0.05*.

*sNAb, surrogate neutralizing antibody*.

## Discussion

We studied a humoral immune response after receiving the CoronaVac vaccine among adult dermatological patients receiving systemic immunosuppressive therapies. According to the Infectious Diseases Society of America definition of immunosuppression, these patients are categorized as having low-level immunosuppression (8). Suboptimal immune response to the vaccine was observed in a subset of participants treated with azathioprine, cyclosporin, mycophenolate mofetil, and moderate-to-high dose prednisolone (≥10 mg/d) during vaccination; in which the majority of these participants are pemphigus patients. Meanwhile, the vaccine can induce an appreciable level of immune response in participants who used biologics (i.e., secukinumab, ixekizumab, and omalizumab), low-dose methotrexate (≤10 mg/d), and low-dose systemic corticosteroids (<10 mg/d); equivalent to controls without immune-mediated skin diseases.

Previous studies investigating CoronaVac's immunogenicity in immunosuppressed patients have yielded conflicting results. For instance, Pestana et al. demonstrated a clinically insignificant seroconversion rate of 15.2% among kidney transplant recipients (9), while Karacin et al. found that more than half of cancer patients receiving chemotherapy were able to seroconvert (10). In patients with autoimmune diseases, the vaccine has demonstrated a reduced yet acceptable level of immune response among ARD patients in which 56.3% of them developed detectable neutralizing antibodies post-vaccination without statistically significant difference in neutralizing activities between ARD patients and healthy controls who seroconverted (4). Our study also observes the same trend in psoriasis patients. By contrast, another cohort study of patients with various immune-mediated diseases found a significant number of patients with low SARS-CoV2 specific antibody titers (5) despite a substantial overlap of immunosuppressants used by participants of this study and the one mentioned above. A similar finding is noticeable among pemphigus patients in our study, as a majority of those who received systemic immunosuppressive therapies did not develop seroconversion. These data suggest that the interaction between hosts' comorbidities and their treatment, rather than individual factors, determines humoral immune responses to inactivated COVID-19 vaccines, resulting in the discrepancy in vaccine response patterns between patients with different immune-mediated diseases. Therefore, for immunocompromised patients to achieve an effective response to inactivated COVID-19 vaccine, it is imperative to evaluate the immunogenicity and efficacy of the vaccine in the context of the specific patient groups of interest.

The main limitation of this study is the small sample size and heterogeneity of the patients recruited; hence, the observed trends from our research should be confirmed by larger studies. Besides, the cellular immune response was not assessed; however, as the previous study of the vaccine has demonstrated a low cellular response in healthy volunteers (11), the same or worse can be expected among these patients. Moreover, the immunosuppressive effects caused by the medications (especially rituximab) extend beyond the drug half-life, further complicated by its distinctive actions on specific B cell subpopulations which was not thoroughly assessed by this study. There is also a lack of participants who received methotrexate > 10 mg/week in this study; immunogenicity in this setting may either be below or equivalent to patients who received methotrexate ≤ 10 mg/week. Further studies with a more variety of medications and detailed lymphocyte subset characterization may uncover a more intricate vaccine response pattern among these patients.

## Conclusion

We present immunogenicity data of the CoronaVac vaccine in a specific target group of dermatological patients who used immunosuppressive therapies. Currently, there are only a few studies that investigate immunogenicity of the vaccine in these patients. The identification of the poor responders is crucial as they could benefit from vaccine platforms that trigger a greater level of immunogenicity. They may require booster doses using COVID-19 vaccines with adequate safety data in immunocompromised patients (12, 13). Further studies are needed to explore the effects of individual immunosuppressive medications and the immune responses in patients with other autoimmune skin diseases not presented in this study (e.g., bullous pemphigoid, dermatomyositis, and vitiligo).

## Data Availability Statement

The original contributions presented in the study are included in the article/supplementary material, further inquiries can be directed to the corresponding author.

## Ethics Statement

The studies involving human participants were reviewed and approved by the Human Research Ethics Committee, Faculty of Medicine Ramathibodi Hospital, Mahidol University. The patients/participants provided their written informed consent to participate in this study.

## Author Contributions

CS-a, CP, and YR conducted the investigation. CS-a collected and prepared the samples, performed data curation, data visualization, formal analysis, and wrote the original draft of the manuscript. CS-a and TS provided laboratory resources and performed the experiments. KC, PR, and PS supervised the project administration. PS and KC acquired funding support for the project and revised the manuscript. All authors involved in the conceptualization, methodology planning of the study, and approved the final version of the manuscript.

## Funding

This study was supported by Mahidol University's Specific League Fund.

## Conflict of Interest

The authors declare that the research was conducted in the absence of any commercial or financial relationships that could be construed as a potential conflict of interest.

## Publisher's Note

All claims expressed in this article are solely those of the authors and do not necessarily represent those of their affiliated organizations, or those of the publisher, the editors and the reviewers. Any product that may be evaluated in this article, or claim that may be made by its manufacturer, is not guaranteed or endorsed by the publisher.
